# Predicting response to chemoradiotherapy in rectal cancer via visual morphologic assessment and staging on baseline MRI: a multicenter and multireader study

**DOI:** 10.1007/s00261-023-03961-7

**Published:** 2023-06-26

**Authors:** Najim El Khababi, Regina G. H. Beets-Tan, Renaud Tissier, Max J. Lahaye, Monique Maas, Luís Curvo-Semedo, Raphaëla C. Dresen, Stephanie Nougaret, Geerard L. Beets, Doenja M. J. Lambregts, on behalf of the rectal MRI study group, Frans C. H. Bakers, Perla Barros, Ferdinand Bauer, Shira H de Bie, Stuart Ballantyne, Joanna Brayner Dutra, Laura Buskov, Nino Bogveradze, Gerlof P. T. Bosma, Vincent C Cappendijk, Francesca Castagnoli, Sotiriadis Charalampos, Andrea Delli Pizzi, Michael Digby, Remy W. F. Geenen, Joost J. M. van Griethuysen, Julie Lafrance, Vandana Mahajan, Sonaz Malekzadeh, Peter A Neijenhuis, Gerald M Peterson, Indra Pieters, Niels W. Schurink, Ruth Smit, Cornelis J. Veeken, Roy F. A. Vliegen, Andrew Wray, Abdel-Rauf Zeina

**Affiliations:** 1 Department of Radiology, The Netherlands Cancer Institute, P.O. Box 90203, 1106 BE Amsterdam, The Netherlands; 2 GROW School for Oncology & Developmental Biology, University of Maastricht, Maastricht, The Netherlands; 3 Biostatistics Unit, The Netherlands Cancer Institute, Amsterdam, The Netherlands; 4 Department of Radiology, Faculty of Medicine, Centro Hospitalar e Universitario de Coimbra EPE, University of Coimbra, Coimbra, Portugal; 5 Department of Radiology, University Hospitals Leuven, Leuven, Belgium; 6 Medical Imaging Department, Montpellier Cancer Institute, Montpellier Cancer Research Institute (U1194), University of Montpellier, Montpellier, France; 7 Department of Surgery, The Netherlands Cancer Institute, Amsterdam, The Netherlands

**Keywords:** Rectal cancer, Magnetic resonance imaging, Chemoradiotherapy, Response

## Abstract

**Purpose:**

Pre-treatment knowledge of the anticipated response of rectal tumors to neoadjuvant chemoradiotherapy (CRT) could help to further optimize the treatment. Van Griethuysen et al. proposed a visual 5-point confidence score to predict the likelihood of response on baseline MRI. Aim was to evaluate this score in a multicenter and multireader study setting and compare it to two simplified (4-point and 2-point) adaptations in terms of diagnostic performance, interobserver agreement (IOA), and reader preference.

**Methods:**

Twenty-two radiologists from 14 countries (5 MRI-experts,17 general/abdominal radiologists) retrospectively reviewed 90 baseline MRIs to estimate if patients would likely achieve a (near-)complete response (nCR); first using the 5-point score by van Griethuysen (1=highly unlikely to 5=highly likely to achieve nCR), second using a 4-point adaptation (with 1-point each for high-risk T-stage, obvious mesorectal fascia invasion, nodal involvement, and extramural vascular invasion), and third using a 2-point score (unlikely/likely to achieve nCR). Diagnostic performance was calculated using ROC curves and IOA using Krippendorf’s alpha (*α*).

**Results:**

Areas under the ROC curve to predict the likelihood of a nCR were similar for the three methods (0.71–0.74). IOA was higher for the 5- and 4-point scores (*α*=0.55 and 0.57 versus 0.46 for the 2-point score) with best results for the MRI-experts (*α*=0.64-0.65). Most readers (55%) favored the 4-point score.

**Conclusions:**

Visual morphologic assessment and staging methods can predict neoadjuvant treatment response with moderate–good performance. Compared to a previously published confidence-based scoring system, study readers preferred a simplified 4-point risk score based on high-risk T-stage, MRF involvement, nodal involvement, and EMVI.

**Graphical abstract:**

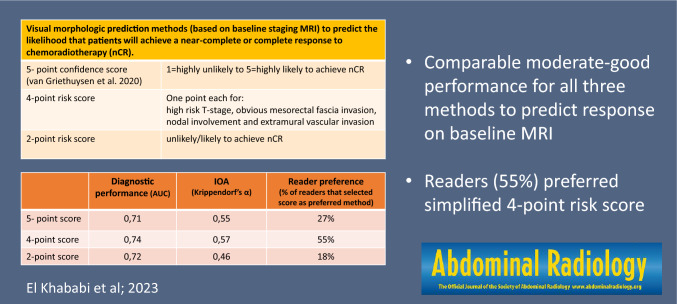

**Supplementary Information:**

The online version contains supplementary material available at 10.1007/s00261-023-03961-7.

## Introduction

Recently, there has been a shift toward more organ-preserving treatments for rectal cancer. Patients with advanced tumors who show clinical evidence of a complete response (CR) after neoadjuvant chemoradiotherapy (CRT) may be entered into a watch-and-wait (W&W) program while patients with small tumor remnants may be cured with local treatment options such as transanal excision instead of major resection [1–3]. In addition, there are ongoing trials (such as the STAR-TREC trial) investigating the benefit of giving chemoradiotherapy to early-stage tumors with the aim of achieving organ preservation [4]. According to current guidelines, these early tumors are typically managed with direct surgery. These developments have urged the need to accurately monitor response after CRT, but have also given rise to an increased interest to predict treatment response before the start of CRT. If we could differentiate at baseline which patients are likely to respond well and which patients will be non-responders, this could aid in the selection of patients who would be good candidates for CRT while avoiding unnecessary side effects in anticipated non-responders. Pre-treatment knowledge of the anticipated treatment response could also help to optimize the neoadjuvant treatment strategies further.

Several studies have investigated the potential role of imaging and image biomarkers as pre-treatment predictors of response [5–9]. So far, these studies have mainly focused on functional imaging techniques such as diffusion-weighted imaging (DWI) and dynamic contrast-enhanced (DCE) MRI, and on multiparametric imaging models developed using artificial intelligence (AI) methods such as radiomics [10–14]. Interestingly, several of these reports have shown that basic tumor descriptors such as the T- and N-stage, morphology, and volume were among the variables showing the best potential to predict response [15, 16].This indicates that visual morphologic interpretation by radiologists is not only crucial for staging but could also be helpful to render predictors of treatment response. Van Griethuysen et al. were one of the first to develop a method to estimate the likelihood of response based solely on radiologists' visual interpretation and staging of baseline MRI scans [17]. They showed that a confidence scoring system taking into account the size, signal, and shape of the tumor, T- and N-stage, mesorectal fascia (MRF) involvement, and extramural vascular invasion (EMVI) could predict the chance of achieving a good or complete response to CRT on baseline MRI with areas under the curve (AUCs) of 0.67-0.83, when assessed by two expert radiologists. To the best of our knowledge, visual morphologic response prediction methods such as the one proposed by van Griethuysen have not yet been evaluated by larger groups of readers and/or using multicenter MRI data.

This study therefore aims to evaluate the visual response prediction method of van Griethuysen et al. in a multicenter study setting and to compare it to two simplified adaptations of the same scoring system in terms of diagnostic performance and reproducibility among a large inter-national group of radiologists with varying levels of expertise.

## Materials and methods

### Patient selection

This retrospective diagnostic study was conducted as part of an ongoing institutional review board approved multicenter project focused on MRI for rectal tumor risk and response assessment, including the imaging and clinical outcome data of 1037 rectal cancer patients from ten centers in the Netherlands acquired between 2010 and 2018. For the current study, we identified from this cohort patients fulfilling the following inclusion criteria: (a) biopsy-proven non-mucinous rectal adenocarcinoma, (b) neoadjuvant treatment consisting of “routine” long course CRT (50.0-50.4 Gy with concurrent capecitabine-based chemotherapy), (c) availability of diagnostic quality primary staging MRI including at least T2-weighted sequences in three planes (sagittal, coronal, transversal), and (d) availability of a final response outcome (histology after surgery or ≥2 years clinical follow-up in case of W&W treatment). From this group, we semi-randomly selected a sample of *n*=90 patients to be included in the current study cohort, taking into consideration that data of all 10 study centers had to be represented and ensuring a clinically representative sample in terms of response outcomes to allow meaningful statistical analyses. This semi-random (selective) approach was chosen, because two of the ten centers are referral centers for W&W, which could have otherwise resulted in relative overrepresentation of complete responders in the cohort. Because of the retrospective nature of the study, informed consent was waived.

### MR imaging

All MRIs were performed according to the local protocols of the participating centers at the time of inclusion. From the full protocols, we selected for this study the 2D T2-weighted spin echo sequences in sagittal, oblique-axial (perpendicular to the tumor axis), and oblique-coronal (parallel to the tumor axis) planes, in line with the minimal requirements for primary rectal cancer staging as outlined in recent guidelines [18]. Slice thickness ranged between 3 and 5 mm and in plane resolution between 0.35x0.35 and 0.94x0.94 mm.

### Image evaluation

MRIs were assessed by twenty-two radiologists from 14 different countries, including five rectal MRI-experts (each with ≥10 years’ dedicated experience in rectal MRI and rectal cancer research) and 17 abdominal radiologists or general radiologists with a specific interest in abdominal imaging. The 17 abdominal/general radiologists had a median of 6 years’ experience in reading rectal MRI (range 1.5–21 years) with an estimated median of 100 (range 50–250) rectal MRI cases read on a yearly basis. Study readers were recruited via an open call to members of the European Society of Gastrointestinal and Abdominal Radiology (ESGAR), in specific those with an interest in rectal imaging. Image evaluation was performed using an in-house developed web-based platform (iScore) that was designed by one of the authors (N.E.K.) and incorporates the Open Health Imaging Foundation (OHIF) DICOM viewing platform [19].

**Fig. 1:**
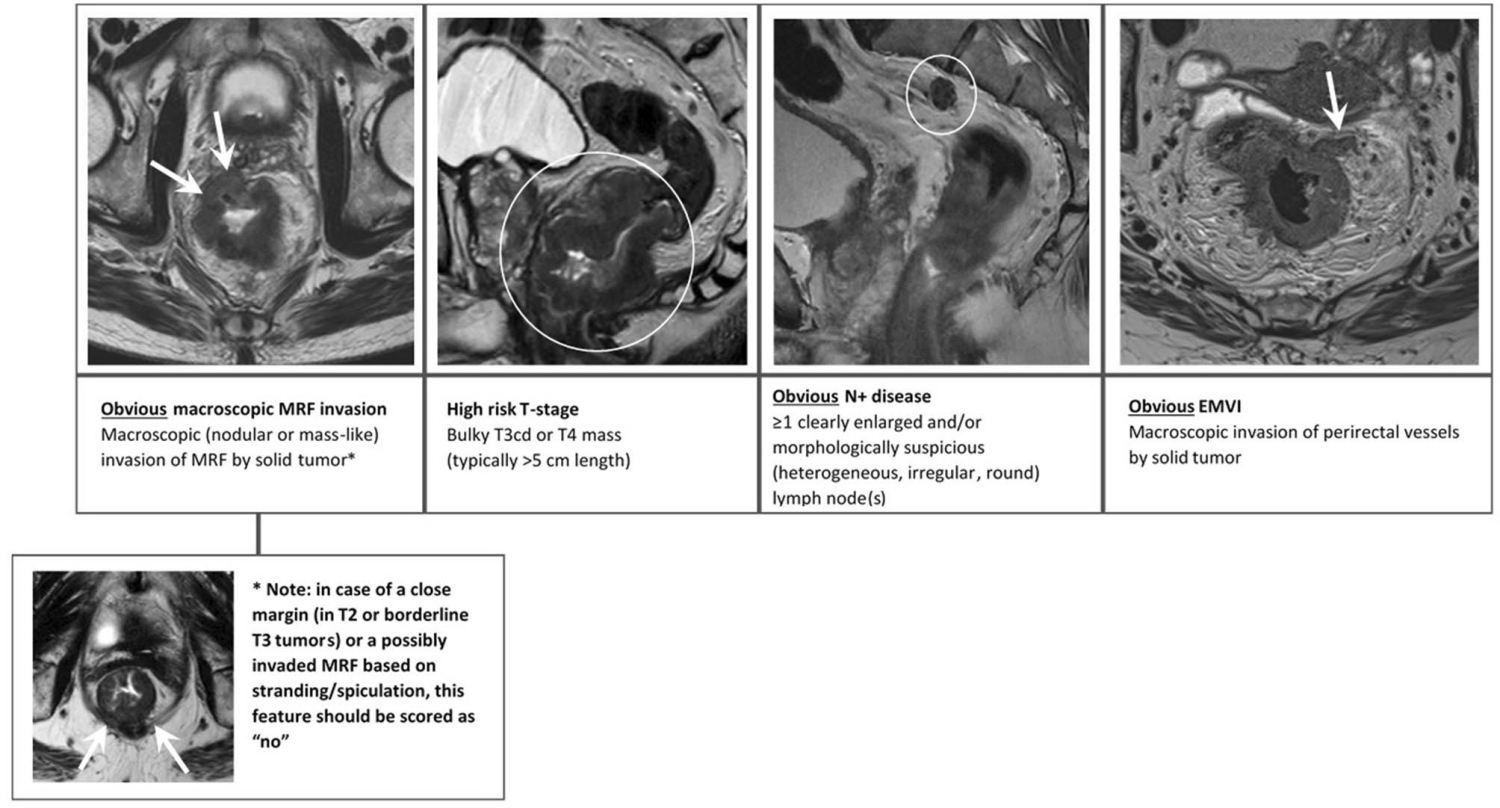
Instructions provided to the study readers to assign a 4-point risk score based on the presence/absence of 4 key high-risk features: obvious MRF invasion, high-risk T-stage (bulky/irregular, T3c-4), obvious node-positive disease, and obvious EMVI. Readers were instructed to only select yes if they were confident that a respective worrisome feature was present. When in doubt, readers were instructed to select ‘no’

**Fig. 2:**
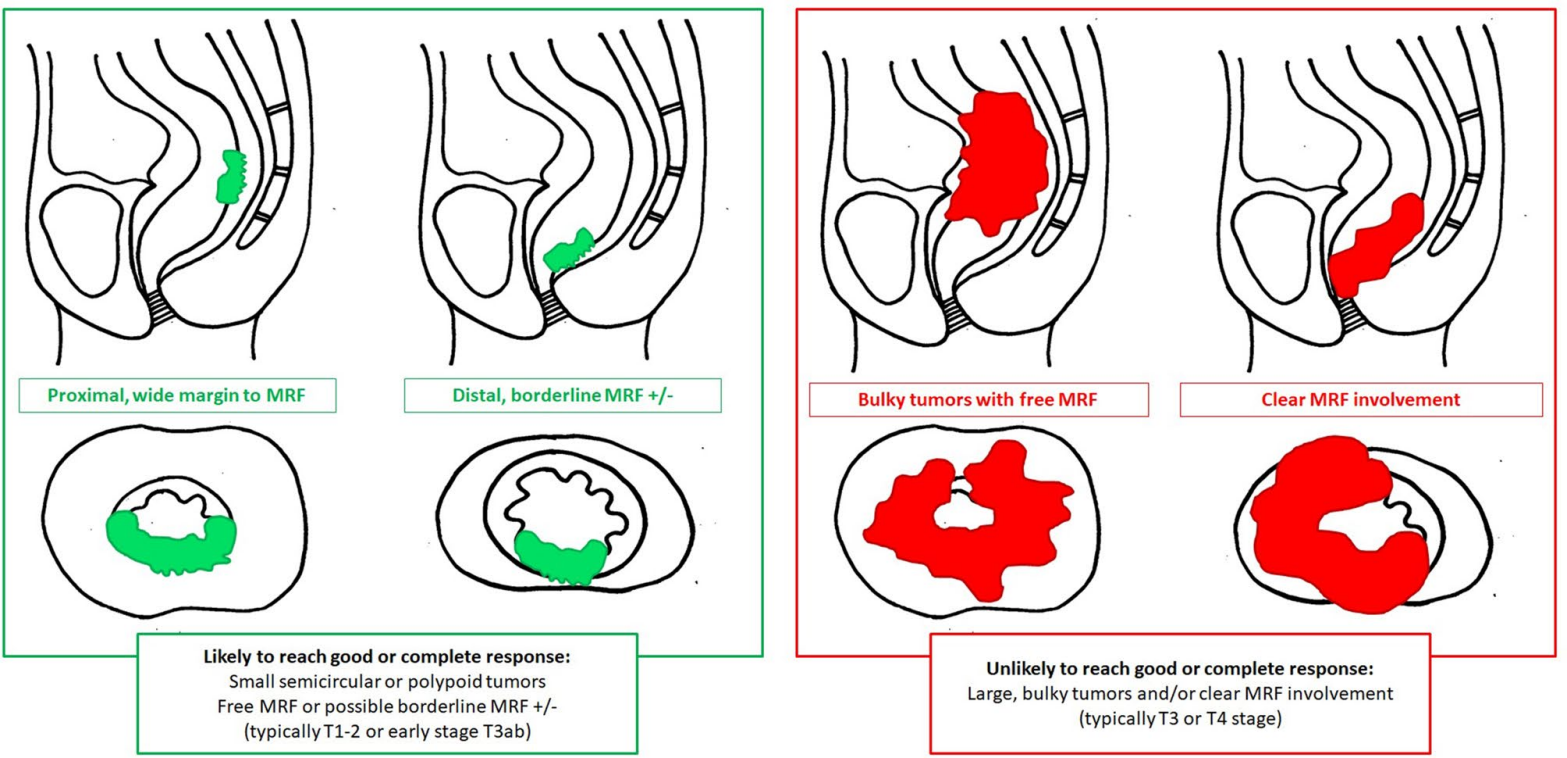
Instructions provided to study readers to assign a dichotomized (2-point) risk score. Green = low risk, i.e., tumor likely to achieve a (near-)complete response. Red = high risk, i.e., tumor unlikely to achieve a (near-)complete response

Study readers were asked to review the baseline MRIs of the 90 study cases using electronic case report forms (eCRFS) that were embedded into iScore. These eCRFs included three different scoring methods designed to estimate the likelihood that patients would achieve a complete or near-complete response to chemoradiotherapy based on the overall tumor risk profile. The first scoring method was the 5-point confidence score published by van Griethuysen et al. that is based on a combination of tumor size, signal heterogeneity, shape (regular/irregular), T-stage, N-stage, EMVI and MRF invasion [17]. The second scoring method was a simplified 4-point adaptation, taking into account only MRF invasion, high-risk T-stage, EMVI, and N-stage. The final scoring method was a further simplified, dichotomized (2-point score) adaptation. Full details of the three scoring methods are provided in Table 1 and supporting images are provided in Figs. 1 and 2. A visual representation of the scoring setup in iScore including the full eCRFS is provided in Supplement 1. Readers were asked to indicate for each individual case whether they found the respective scoring methods easy, moderately easy/difficult, or difficult to apply. Finally, after completion of all cases, they were asked to give an overall indication of which scoring method they would prefer to use in their daily clinical practice. Readers were blinded to each other’s scorings and to the final response outcomes.

**Table 1:** Scoring methods used to predict response to chemoradiotherapy on baseline MRI

Response method	Score
5-point confidence score (van Griethuysen et al.)*	1 = High-risk—Highly unlikely to achieve (near-)complete response (7 high-risk criteria)* 2 = Moderately high-risk—Unlikely to achieve (near-)complete response (≥5 high-risk criteria)* 3 = Intermediate risk—Equivocal (Not meeting the criteria for scores 1–2 or 4–5) 4 = Moderately low risk—Likely to achieve (near-)complete response (≥3 low risk criteria)* 5 = Low risk—Highly likely to achieve (near-)complete response (≥5 low risk criteria)*
4-point risk score (see Fig. 1)	1-point for each of the following high-risk features (total score 0-4): - Obvious macroscopic MRF invasion^#^ - High-risk T-stage (bulky, T3c-4)^#^ - Obvious nodal involvement^#^ - Obvious EMVI^#^
Dichotomized (2-point) risk score (see Fig. 2)	0 = **Likely to reach good or complete response:** - Small semicircular or polypoid tumors - Free MRF (or possibly borderline MRF involvement) - T1-2 or early-stage T3 (T3ab) 1 = **Unlikely to reach good or complete response:** - Bulky semicircular or circular tumors - High-risk (T3cd or T4) T-stage - With or without MRF involvement

*The confidence score of van Griethuysen is a composite score that combines T-stage, size, signal (heterogeneous/homogeneous), shape (regular/irregular), N-stage, EMVI, and MRF involvement as assessed on T2-weighted MRI. High-risk criteria include ≥T3cd stage, tumor size > 5 cm, heterogeneous tumor signal, irregular tumor shape, N+ stage, EMVI+, and MRF+. Low risk criteria include ≤T3ab stage, tumor size < 3 cm, homogeneous tumor signal, regular tumor shape, N0 stage, EMVI- and MRF-. Readers were provided with the originally published paper and scoring definitions while performing their scorings [17]

#Readers were instructed to only indicate ‘yes’ if they were confident that a respective high-risk feature was present. When in doubt, they were instructed to select ‘no’

### Standard of reference

The main outcome of this study was to predict a (near-)complete response, which was defined as the absence of viable cancer cells, or the presence of only rare or small clusters of residual cancer cells at histopathology after surgery. The primary standard of reference in the patients that had undergone surgery was the histopathological Mandard tumor regression grade (TRG), where a (near-)complete response was defined as TRG 1-2 [20]. In patients undergoing W&W, a sustained clinical complete response with a local regrowth-free follow-up period of ≥2 years was considered a surrogate endpoint of a complete response (TRG1).

### Statistical analysis

Statistical analyses were performed using R statistics version 4.1.0 (2021) and IBM SPSS version 27 (2020). The scores from the 22 radiologists were averaged for each patient in order to produce a probability of response that was then used to compute Receiver Operator Characteristics (ROC) curves and calculate mean areas under the curve (AUC) for each scoring method. Optimal cut-off values for the 5-point and 4-point scores were derived from the ROC curves to calculate sensitivity, specificity, positive predictive value (PPV), negative predictive value (NPV), and overall accuracy to predict a (near-)complete response (being the positive study outcome). Results were separately analyzed for the five MRI-experts versus the 17 less experienced readers, and mixed model linear regression was used to assess the impact of reader experience on the diagnostic accuracy figures of each scoring method. To account for the repeated measurements of each patient, a patient-level random intercept was used. A logistic regression was performed to analyze the possibility of an association between the diagnosis accuracy and the interval between completion of CRT and final surgery/entry into a W&W program. To do so, the proportion of correct diagnoses for each patient and method was computed across all readers. This proportion was then used as response and interval between completion of CRT and final surgery/entry into a W&W program was used as a covariate. *p*-values <0.05 were considered statistically significant. Group interobserver agreement (IOA) was calculated using Krippendorff’s alpha (*α*).

## Results

### Patient characteristics

Table 2 shows the baseline characteristics of the 90 study patients. Fifty-two patients (58%) were male; median age was 65 years (range 41–82). Forty-four patients (49%) were (near-)complete responders, including 27 (30%) complete responders (21 after surgery; 6 clinical complete responders undergoing W&W). Mean time interval between completion of CRT and surgery (or inclusion into a W&W program) was 11±2.5 weeks.

**Table 2:** Patient characteristics

		*N*=	%
Patients	*N*=90	90	100
Median age (ranges)	64.5 (41-82)		
Sex	Male	52	58
	Female	38	42
Baseline clinical stage as reported on MRI			
cT-stage	cT1-2	3	3
	cT3	68	76
	cT4	18	20
cN-stage	cN0	12	13
	cN1	19	21
	cN2	59	66
Final response			
yT-stage	yT0*	27	30
	yT1-2	22	24
	yT3	37	41
	yT4	4	4
yN-stage	yN0*	65	72
	yN1	17	19
	yN2	8	9
TRG	TRG1*	27	30
	TRG2	17	19
	TRG3	29	32
	TRG4	15	17
	TRG5	2	2
Response categorization	(Near-)complete response (TRG1-2)*	44	49
	Incomplete response (TRG3-5)	46	51

*Based on histology after surgery in 21 patients and on a sustained clinical complete response during W&W with >2 years of clinical follow-up in the remaining 6 patients

### Diagnostic performance to predict a (near-)complete response

Average performance for all study readers to predict a (near-)complete response to CRT was similar for all three methods with an AUC of 0.71 (95% CI 0.60–0.82) for the 5-point confidence score, AUC 0.74 (95% CI 0.64–0.84) for the 4-point risk score, and AUC 0.72 (95% CI 0.62–0.83) for the dichotomized 2-point risk score; differences in AUC between the three methods were not statistically significant (*p*=0.10–0.64). Further accuracy figures are provided in Table 3. The 5-point confidence score resulted in slightly lower sensitivity than the other two methods (49% versus 57-59%); the other metrics were similar for the three different scoring methods. There was a tendency toward higher performance for the MRI-experts versus less expert readers, though these differences did not reach statistical significance (*p*=0.15–0.99; except for the PPV of the 5-point confidence score where the MR-experts scored significantly higher than the non-experts, *p*=0.03). The time interval between CRT and surgery/W&W had a significant confounding effect (with a tendency toward higher performance with longer intervals).

**Table 3:** Diagnostic performance and effect of reader experience level

			Sensitivity		Specificity		PPV		NPV		Accuracy		AUC	
5-point confidence score	Average (all readers)		49%		73%		66%		62%		61%		0.71	
	Expert readers	Non-expert readers	43%	51%	83%	70%	72%	64%	62%	62%	63%	61%	0.75	0.69
	Effect size (+ 95% CI)		− 0.08 (− 0.32;0.16)		0.13 (− 0.06;0.31)		0.08 (− 0.01;0.15)		0.00 (− 0.08;0.08)		0.03 (− 0.02;0.07)		N/A	
	Level of significance (*p*)		*p*=0.49		*p*=0.17		*p*=0.03		*p*=0.99		*p*=0.27		*p*=0.16	
4-point risk score	Average (all readers)		57%		71%		67%		65%		64%		0.74	
	Expert readers	Non-expert readers	59%	57%	74%	70%	69%	66%	67%	64%	67%	64%	0.76	0.74
	Effect size (+ 95% CI)		0.02 (− 0.17;0.21)		0.04 (− 0.11;0.20)		0.02 (− 0.05;0.09)		0.03 (− 0.04;0.10)		0.03 (− 0.03;0.09)		N/A	
	Level of significance (*p*)		*p*=0.81		*p*=0.59		*p*=0.51		*p*=0.42		*p*=0.28		*p*=0.38	
Dichotomized (2-point) risk score	Average (all readers)		59%		68%		64%		64%		64%		0.72	
	Expert readers	Non-expert readers	60%	58%	71%	68%	67%	64%	66%	64%	66%	63%	0.74	0.71
	Effect size (+ 95% CI)		0.01 (− 0.13;0.16)		0.04 (− 0.08;0.15)		0.03 (− 0.01;0.08)		0.02 (− 0.04;0.07)		0.02 (− 0.02;0.07)		N/A	
	Level of significance (*p*)		*p*=0.86		*p*=0.51		*p*=0.15		*p*=0.49		*p*=0.24		*p*=0.39	

Results were calculated using a (near-)complete response (TRG1-2) as the positive outcome and incomplete response as the negative outcome. Expert readers (*n*=5) were MRI-experts with ≥10 years dedicated experience in rectal MRI; non-expert readers (*n*=17) were abdominal radiologists or general radiologists with a specific interest in abdominal imaging. Effect sizes for reader experience level including 95% confidence intervals and levels of significance were assessed using mixed model linear regression.

Optimal cut-off values were derived from the results of the ROC-analysis: the 5-point confidence score by van Griethuysen was dichotomized between 4 and 5 (positive, indicative of (near-)CR) and 1-3 (negative, indicative of incomplete response); the 4-point risk score was dichotomized between 0-1 (positive, indicative of (near-)CR) and 2-4 (negative, indicative of incomplete response)

### Interobserver agreement and reader preference

Table 4 shows the interobserver agreement for the three scoring methods, including specified results for the expert and non-expert readers. Table 5 shows the reader feedback (i.e., perceived difficulty per case and overall preferred scoring methods). Group IOA (Krippendorff’s alpha) for all readers combined was similar for the 5-point confidence level score (*α*=0.55) and the 4-point risk score (*α*=0.57), and lower for the 2-point score (*α*= 0.46). Agreement was higher for the MRI-experts compared to the less experienced readers, especially for the 5-point confidence score (*α*=0.64 versus 0.53) and for the 4-point risk score (*α*=0.65 versus 0.55). When looking at the individual variables included in the 4-point risk score, IOA for the assessment of EMVI and MRF involvement was higher than for the assessment of high-risk T-stage and nodal involvement. Most readers found the simplified 4-point and 2-point risk scores easier to apply, compared to the 5-point confidence level score; most readers (55%) selected the 4-point risk score as their preferred method of response prediction.

**Table 4:** Interobserver agreement (Krippendorf’s alpha)

	All readers (*n*=22)	Expert readers (*n*=5)	Non-expert readers (*n*=17)
5-point confidence score	0.55	0.64	0.53
4- point risk score (Total)	0.57	0.65	0.55
- MRF invasion	0.47	0.60	0.45
- High risk (bulky, T3cd-4) T-stage	0.39	0.39	0.39
- Nodal involvement	0.37	0.43	0.34
- EMVI	0.46	0.54	0.44
2-point risk score	0.46	0.44	0.47

**Table 5:** Reader preference

Difficulty to apply method (%)*	5-point confidence score (%)	4- point risk score (%)	2-point risk score (%)
Easy	52	69	74
Moderate	40	30	21
Difficult	7	2	5
Preferred method (%)**	27	55	18

*Scored for each individual case (percentages represent the average scores for all cases and readers combined)

**Scored once, after completion of all study cases (percentages represent the average scores for all readers combined)

## Discussion

With this study, we investigated the value MRI to estimate the chance that patients will undergo a (near-)complete response to neoadjuvant chemoradiotherapy based on visual morphologic risk assessment and staging performed on baseline MRI. A previously published 5-point confidence score and two simplified (4-point and 2-point) adaptations were tested and compared in terms of diagnostic performance, interobserver reproducibility, and reader preference. Diagnostic performance to predict a (near-)complete response was similar for the three methods with AUCs ranging between 0.71 and 0.74. When also considering interobserver agreement and reader preference, a 4-point risk score based on a combination of high-risk T-stage, MRF invasion, EMVI, and nodal involvement showed the most favorable results.

Interobserver agreement in our study was at best moderate (*α*=0.46–0.57), with somewhat better results for the MRI-experts, especially for the 4- and 5-point scores (*α*=0.64–0.65). The more expert radiologists also showed a tendency toward better diagnostic performance, albeit that the difference in performance did not reach statistical significance in most cases. IOA for the most simplified 2-point risk score was similarly low for the experts and non-experts (*α*=0.44–0.47). The previously published confidence score proposed by van Griethuysen is a relatively complex composite score that incorporates T-stage, size, signal, shape, N-stage, EMVI, and MRF involvement. We hypothesized that by simplifying this score, we might be able to improve the interreader reproducibility. However, such an effect was not observed. Nevertheless, most readers did show a clear preference for the simplified scoring systems—in particular the 4-point risk score—and indicated that they found this method more straightforward to apply. This scoring system may therefore be more easy to adapt in daily practice, especially by more general readers.

Specificity to predict patients unlikely to achieve a (near-)complete response was relatively high (ranging between 68% and 73%) and considerably higher than the sensitivity of only 49%–59% to predict which patients would become (near-)complete responders. These results indicate that the study readers were better at estimating patients likely to end up with residual tumor. We hypothesize that recognizing the really “ugly” tumor cases (unlikely to ever reach organ preservation) may be relatively straightforward, while there is a more broad spectrum of “intermediate risk” cases where it will be more challenging to predict which patients will proceed to show a good response versus a (near-) complete response to treatment. Interestingly, our results are also more or less in line with previous reports on assessing response in the restaging setting after completion of CRT where radiologists are generally also better at identifying poor responders than in identifying complete (or near-complete) responders [21–23]. Ultimately, the selection of patients for organ preservation should not be based on imaging only, but informed by a combination of MRI, clinical (digital rectal) examination, and endoscopy [3, 24].

Of note, our current results are based solely on “simple” visual morphologic assessment and baseline staging of anatomical MR images by radiologists, without the need for additional quantitative measurements, advanced (functional) imaging sequences, or computational algorithms. The benefit of such an approach is that it can easily be implemented in daily practice and is relatively comprehensive for clinicians. An important drawback, however, is that it is also observer dependent and influenced by the experience level of radiologists, as also reflected by our results that show a tendency toward higher IOA and diagnostic performance for the more experienced study readers. Though we aimed to provide readers with clear scoring instructions (see Figs. 1 and 2), criteria such as ‘obvious nodal involvement’ and ‘bulky tumor’ remain subjective criteria, which probably contributed to the relatively low IOA. These effects are less of an issue when using more quantitative or AI-based methods, which have formed a major topic of research in recent literature. Functional imaging parameters such as the Apparent Diffusion Coefficient (ADC) derived from diffusion-weighted MRI, and perfusion metrics (e.g., K-trans) derived from dynamic contrast-enhanced MRI, have all shown potential as pre-treatment predictors of response [5, 25]. In addition, “texture” features such as entropy and uniformity that reflect tissue heterogeneity have been associated with the chance of successful tumor response [15, 16, 26]. When combining such quantitative features in multivariable (radiomics) models, published reports have shown varying AUCs ranging between 0.68 and 0.97 to predict rectal tumor response at baseline [27]. Van Griethuysen et al. showed that the predictive performance of a quantitative AI model was similar to that of a visual morphologic response prediction performed by experienced radiologists [17]. Other studies have shown a complementary value for AI (radiomics) and visual morphologic evaluations and have demonstrated that combining these two approaches can increase diagnostic performance to predict response [17, 27–30]. Nevertheless, reported results for image-based prediction methods (regardless of whether visual morphologic and/or quantitative) are highly variable with AUCs in many reports not exceeding 0.80; performance levels that will likely not be considered sufficient to impact treatment planning. Response to anti-cancer treatment is a multifactorial process that is not only dependent on tumor size and morphology, but also on other patient-factors and aspects of tumor biology that we cannot hope to capture by imaging. Important next steps in research will therefore be to combine image-based prediction methods with other clinical, histopathological, immunohistochemical and genetic biomarkers that have shown promise as predictors of response and that were unfortunately not available for analysis in this current retrospective study cohort [27, 29–33]. Only this way can we hope to achieve a strong enough predictive performance to serve as a basis for clinical decision-making, aiming to further boost personalized therapy in rectal cancer.

There are some limitations to our study design, in addition to its retrospective nature. To ensure that it would be feasible for a multitude of readers to complete the full set of study cases within an acceptable timeframe, the cohort size was deliberately kept relatively small. We fully acknowledge that the semi-random selection of patients from a larger cohort (ensuring a balanced sample in terms of representation of data from the different participating centers and response outcomes), may be prone to bias though we are confident that our cohort including data from ten different centers offers a representative sample reflective of everyday clinical routine. The study dataset dates back to 2010, which entails that some MRIs were acquired with ‘outdated’ study protocols. Though we acknowledge these variations may have had an impact on overall scan quality, we believe that these effects will likely be limited considering that evaluations were mainly based on routine T2-weighted imaging which will probably show less variation in quality over time than for example DWI. While the 17 less expert readers in our cohort were intended to offer a representative sample of radiologists reading rectal MRI in everyday clinical practice, we cannot rule out a certain selection bias considering that readers were recruited via an open call to ESGAR members (with a specific interest in rectal cancer). Finally, our results should be interpreted with some caution as we have shown that response, and corresponding performance to predict response, was influenced by variations in the interval between CRT and surgery/W&W. Prolonging the interval between CRT and surgery is a known factor that generally results in higher response rates [34–38]. Though variations were small (mean interval between CRT and surgery/W&W was 11 weeks with a standard deviation of 2.5 weeks), a confounding effect could nevertheless not be avoided in this retrospective study setting.

## Conclusions

In conclusion, this multicenter and multireader study has shown that visual morphologic methods to predict response to chemoradiotherapy on baseline staging MRI have a moderate–good diagnostic performance to estimate the likelihood that patients will achieve a (near-)complete response to CRT. Specificity is relatively high, indicating that imaging is mainly good in identifying the more high-risk patients that are unlikely to achieve organ preservation. Overall interobserver agreement is moderate, with better results for more experienced radiologists. Compared to a previously published confidence-based scoring system, study readers preferred a more simplified 4-point risk score based on high-risk T-stage, MRF involvement, nodal involvement, and EMVI. While results are obviously too premature to base clinical decision-making on, they are encouraging and warrant further multidisciplinary research focused on combining imaging with other clinical predictors of response.

## Supplementary Information

Below is the link to the electronic supplementary material.

Supplementary file1 (PDF 839 KB)

## References

[1] Oronsky B, Reid T, Larson C, Knox SJ (2020). Locally advanced rectal cancer: The past, present, and future. Semin Oncol.

[2] Fernandes MC, Gollub MJ, Brown G (2022) The importance of MRI for rectal cancer evaluation. Surg Oncol 43:101739. 10.1016/j.suronc.2022.101739 10.1016/j.suronc.2022.101739 PMC9464708 35339339

[3] van der Valk MJM, Hilling DE, Bastiaannet E (2018). Long-term outcomes of clinical complete responders after neoadjuvant treatment for rectal cancer in the International Watch & Wait Database (IWWD): an international multicentre registry study. Lancet.

[4] Bach SP, STAR-TREC Collaborative (2022). Can we Save the rectum by watchful waiting or TransAnal surgery following (chemo)Radiotherapy versus Total mesorectal excision for early REctal Cancer (STAR-TREC)? Protocol for the international, multicentre, rolling phase II/III partially randomized patient preference trial evaluating long-course concurrent chemoradiotherapy versus short-course radiotherapy organ preservation approaches. Colorectal Dis.

[5] Schurink NW, Lambregts DMJ, Beets-Tan RGH (2019). Diffusion-weighted imaging in rectal cancer: current applications and future perspectives. Br J Radiol.

[6] Ryan JE, Warrier SK, Lynch AC, Heriot AG (2015). Assessing pathological complete response to neoadjuvant chemoradiotherapy in locally advanced rectal cancer: a systematic review. Colorectal Dis.

[7] Hötker AM, Garcia-Aguilar J, Gollub MJ (2014). Multiparametric MRI of rectal cancer in the assessment of response to therapy: a systematic review. Dis Colon Rectum.

[8] Pham TT, Liney GP, Wong K, Barton MB (2017). Functional MRI for quantitative treatment response prediction in locally advanced rectal cancer. Br J Radiol.

[9] Joye I, Deroose CM, Vandecaveye V, Haustermans K (2014). The role of diffusion-weighted MRI and (18)F-FDG PET/CT in the prediction of pathologic complete response after radiochemotherapy for rectal cancer: a systematic review. Radiother Oncol.

[10] Intven M, Monninkhof EM, Reerink O, Philippens ME (2015). Combined T2w volumetry, DW-MRI and DCE-MRI for response assessment after neo-adjuvant chemoradiation in locally advanced rectal cancer. Acta Oncol.

[11] Ippolito D, Fior D, Trattenero C (2015). Combined value of apparent diffusion coefficient-standardized uptake value max in evaluation of post-treated locally advanced rectal cancer. World J Radiol.

[12] Joye I, Debucquoy A, Deroose CM (2017). Quantitative imaging outperforms molecular markers when predicting response to chemoradiotherapy for rectal cancer. Radiother Oncol.

[13] Nie K, Shi L, Chen Q (2016). Rectal Cancer: Assessment of Neoadjuvant Chemoradiation Outcome based on Radiomics of Multiparametric MRI. Clin Cancer Res.

[14] Lambrecht M, Deroose C, Roels S (2010). The use of FDG-PET/CT and diffusion-weighted magnetic resonance imaging for response prediction before, during and after preoperative chemoradiotherapy for rectal cancer. Acta Oncol.

[15] Schurink NW, Min LA, Berbee M (2020). Value of combined multiparametric MRI and FDG-PET/CT to identify well-responding rectal cancer patients before the start of neoadjuvant chemoradiation. Eur Radiol.

[16] Schurink NW, van Kranen SR, Berbee M (2021). Studying local tumour heterogeneity on MRI and FDG-PET/CT to predict response to neoadjuvant chemoradiotherapy in rectal cancer. Eur Radiol.

[17] van Griethuysen JJM, Lambregts DMJ, Trebeschi S (2020). Radiomics performs comparable to morphologic assessment by expert radiologists for prediction of response to neoadjuvant chemoradiotherapy on baseline staging MRI in rectal cancer. Abdom Radiol (NY).

[18] Beets-Tan RGH, Lambregts DMJ, Maas M et al (2018) Magnetic resonance imaging for clinical management of rectal cancer: Updated recommendations from the 2016 European Society of Gastrointestinal and Abdominal Radiology (ESGAR) consensus meeting [published correction appears in Eur Radiol. 2018 Jan 10;:]. Eur Radiol 28(4):1465-1475. 10.1007/s00330-017-5026-2 10.1007/s00330-017-5026-2 PMC5834554 29043428

[19] Ziegler E, Urban T, Brown D (2020). Open Health Imaging Foundation Viewer: An Extensible Open-Source Framework for Building Web-Based Imaging Applications to Support Cancer Research. JCO Clin Cancer Inform.

[20] Mandard AM, Dalibard F, Mandard JC (1994). Pathologic assessment of tumor regression after preoperative chemoradiotherapy of esophageal carcinoma. Clinicopathologic correlations. Cancer.

[21] Haak HE, Maas M, Lahaye MJ (2020). Selection of Patients for Organ Preservation After Chemoradiotherapy: MRI Identifies Poor Responders Who Can Go Straight to Surgery. Ann Surg Oncol.

[22] Seo N, Kim H, Cho MS, Lim JS (2019). Response Assessment with MRI after Chemoradiotherapy in Rectal Cancer: Current Evidences. Korean J Radiol.

[23] van der Sande ME, Beets GL, Hupkens BJ (2019). Response assessment after (chemo)radiotherapy for rectal cancer: Why are we missing complete responses with MRI and endoscopy?. Eur J Surg Oncol.

[24] Maas M, Lambregts DM, Nelemans PJ (2015). Assessment of Clinical Complete Response After Chemoradiation for Rectal Cancer with Digital Rectal Examination, Endoscopy, and MRI: Selection for Organ-Saving Treatment. Ann Surg Oncol.

[25] Dijkhoff RAP, Beets-Tan RGH, Lambregts DMJ, Beets GL, Maas M (2017). Value of DCE-MRI for staging and response evaluation in rectal cancer: A systematic review. Eur J Radiol.

[26] O'Connor JP, Rose CJ, Waterton JC, Carano RA, Parker GJ, Jackson A (2015). Imaging intratumor heterogeneity: role in therapy response, resistance, and clinical outcome. Clin Cancer Res.

[27] Miranda J, Tan GXV, Fernandes MC (2022). Rectal MRI radiomics for predicting pathological complete response: Where we are. Clin Imaging.

[28] Meng Y, Zhang Y, Dong D (2018). Novel radiomic signature as a prognostic biomarker for locally advanced rectal cancer. J Magn Reson Imaging.

[29] Petkovska I, Tixier F, Ortiz EJ (2020). Clinical utility of radiomics at baseline rectal MRI to predict complete response of rectal cancer after chemoradiation therapy. Abdom Radiol (NY).

[30] Cui Y, Yang X, Shi Z (2019). Radiomics analysis of multiparametric MRI for prediction of pathological complete response to neoadjuvant chemoradiotherapy in locally advanced rectal cancer. Eur Radiol.

[31] Smolskas E, Mikulskytė G, Sileika E, Suziedelis K, Dulskas A (2022). Tissue-Based Markers as a Tool to Assess Response to Neoadjuvant Radiotherapy in Rectal Cancer-Systematic Review. Int J Mol Sci.

[32] Lee HH, Chen CH, Huang YH, Chiang CH, Huang MY (2022) Biomarkers of Favorable vs. Unfavorable Responses in Locally Advanced Rectal Cancer Patients Receiving Neoadjuvant Concurrent Chemoradiotherapy. Cells 11(10):1611. 10.3390/cells11101611 10.3390/cells11101611 PMC9139800 35626648

[33] Dizdarevic E, Hansen TF, Jakobsen A (2022). The Prognostic Importance of ctDNA in Rectal Cancer: A Critical Reappraisal. Cancers (Basel).

[34] Sloothaak DA, Geijsen DE, van Leersum NJ (2013). Optimal time interval between neoadjuvant chemoradiotherapy and surgery for rectal cancer. Br J Surg.

[35] Probst CP, Becerra AZ, Aquina CT (2015). Extended Intervals after Neoadjuvant Therapy in Locally Advanced Rectal Cancer: The Key to Improved Tumor Response and Potential Organ Preservation. J Am Coll Surg.

[36] Petrelli F, Sgroi G, Sarti E, Barni S (2016). Increasing the Interval Between Neoadjuvant Chemoradiotherapy and Surgery in Rectal Cancer: A Meta-analysis of Published Studies. Ann Surg.

[37] Du D, Su Z, Wang D, Liu W, Wei Z (2018). Optimal Interval to Surgery After Neoadjuvant Chemoradiotherapy in Rectal Cancer: A Systematic Review and Meta-analysis. Clin Colorectal Cancer.

[38] Awawda M, Taha T, Salman S, Billan S, Hijab A (2022) The evolving treatment paradigm of locally advanced rectal cancer: a narrative review. J Gastrointest Oncol 13(4):2033-2047. 10.21037/jgo-22-13 10.21037/jgo-22-13 PMC9459200 36092339

