# Supplementary material for: Predicting response to chemoradiotherapy in rectal cancer via visual morphologic assessment and staging on baseline MRI: a multicenter and multireader study
Source: Abdom Radiol (NY). 2023 Jun 26;48(10):3039–49. doi: 10.1007/s00261-023-03961-7 (PMC10480283; doi:10.1007/s00261-023-03961-7)
Supplement: Supplementary file 1 — Supplementary file1 (PDF 839 KB) [file 261_2023_3961_MOESM1_ESM.pdf]

Supplement 1

The images below are screenshots derived from the iScore web-based viewing and scoring platform. The first panel (A) shows the overall set up including the Open Health Imaging Foundation (OHIF) DICOM viewing platform on the left and the electronic case report forms (Score Forms) on the right. Panels B-D are detailed views of the three different scoring systems that were embedded in the Score forms. For the 5-point confidence level scoring system previously published by van Griethuysen et al.[17] a link to the scoring instructions derived from the original publication, as well as a PDF link to the full paper were provided as a hyperlink; visual instructions on how to perform the scorings for the two newly developed methods (the 4 point risk score and the dichotomized score) were also embedded as hyperlinks in the Score Forms (red arrows); see also Figures 1 and 2 in the main manuscript.

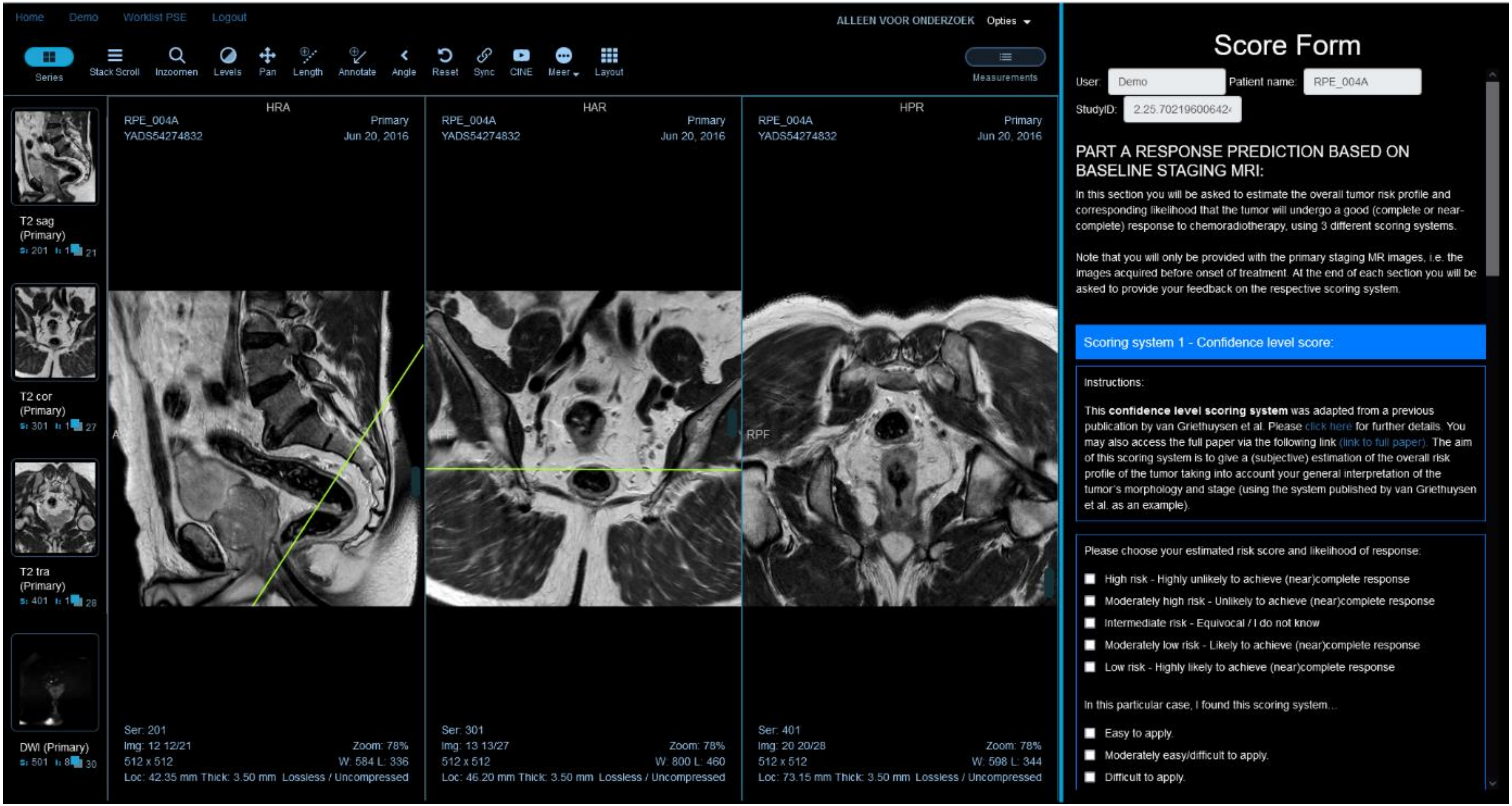
Home
Demo
Worklist PSE
Logout
ALLEEN VOOR ONDERZOEK
Opties
Series
Stack Scroll
Inzoomen
Levels
Pan
Length
Annotate
Angle
Reset
Sync
CINE
Meer
Layout
Measurements
T2 sag (Primary)
T2 cor (Primary)
T2 tra (Primary)
DWI (Primary)
HRA
HAR
HPR
RPE_004A
YADS54274832
Primary
Jun 20, 2016
Ser: 201
Img: 12 12/21
512 x 512
Loc: 42.35 mm Thick: 3.50 mm Lossless / Uncompressed
Zoom: 78%
W: 584 L: 336
Ser: 301
Img: 13 13/27
Loc: 46.20 mm Thick: 3.50 mm Lossless / Uncompressed
W: 800 L: 460
Ser: 401
Img: 20 20/28
Loc: 73.15 mm Thick: 3.50 mm Lossless / Uncompressed
W: 598 L: 344
Score Form
User: Demo
Patient name: RPE_004A
StudyID: 2.25.70219600642
PART A RESPONSE PREDICTION BASED ON BASELINE STAGING MRI:
In this section you will be asked to estimate the overall tumor risk profile and corresponding likelihood that the tumor will undergo a good (complete or near-complete) response to chemoradiotherapy, using 3 different scoring systems.
Note that you will only be provided with the primary staging MR images, i.e. the images acquired before onset of treatment. At the end of each section you will be asked to provide your feedback on the respective scoring system.
Scoring system 1 - Confidence level score:
Instructions:
This confidence level scoring system was adapted from a previous publication by van Griethuysen et al. Please click here for further details. You may also access the full paper via the following link (link to full paper). The aim of this scoring system is to give a (subjective) estimation of the overall risk profile of the tumor taking into account your general interpretation of the tumor's morphology and stage (using the system published by van Griethuysen et al. as an example).
Please choose your estimated risk score and likelihood of response:
High risk - Highly unlikely to achieve (near)complete response
Moderately high risk - Unlikely to achieve (near)complete response
Intermediate risk - Equivocal / I do not know
Moderately low risk - Likely to achieve (near)complete response
Low risk - Highly likely to achieve (near)complete response
In this particular case, I found this scoring system...
Easy to apply.
Moderately easy/difficult to apply.
Difficult to apply.

Panel A

Scoring system 1 - Confidence level score:

Instructions:

This **confidence level scoring system** was adapted from a previous publication by van Griethuysen et al. Please click here for further details. You may also access the full paper via the following link (link to full paper). The aim of this scoring system is to give a (subjective) estimation of the overall risk profile of the tumor taking into account your general interpretation of the tumor's morphology and stage (using the system published by van Griethuysen et al. as an example).

Please choose your estimated risk score and likelihood of response:

- [ ] High risk - Highly unlikely to achieve (near)complete response
- [ ] Moderately high risk - Unlikely to achieve (near)complete response
- [ ] Intermediate risk - Equivocal / I do not know
- [ ] Moderately low risk - Likely to achieve (near)complete response
- [ ] Low risk - Highly likely to achieve (near)complete response

In this particular case, I found this scoring system...

- [ ] Easy to apply.
- [ ] Moderately easy/difficult to apply.
- [ ] Difficult to apply.

Panel B

Scoring system 2 - Simplified 4-point risk score:

Instructions:

The aim of this **4-point scoring system** is to assign a risk score based on the presence/absence of 4 key worrisome features: obvious MRF invasion, bulky/irregular tumor morphology, obvious node-positive disease and obvious EMVI. The following link explains these 4 features and how to apply them for this scoring system. Note, only indicate 'yes' if you are confident that a respective worrisome feature is present. When in doubt, select 'no'.

Please indicate whether the following key high risk features are present or absent in the current case:

- Obvious macroscopic MRF invasion: [ ] Yes [ ] No

- Bulky and/or irregular tumor: [ ] Yes [ ] No

- Obvious nodal involvement: [ ] Yes [ ] No

- Obvious EMVI: [ ] Yes [ ] No

TOTAL SCORE:

In this particular case, I found this scoring system...

- [ ] Easy to apply.
- [ ] Moderately easy/difficult to apply.
- [ ] Difficult to apply.

Panel C

Scoring system 3 - Pattern-based dichotomized score:

Instructions:

The aim of this **pattern-based dichotomized scoring system** is to classify patients as either:

Green = low risk, i.e. tumor likely to achieve a (near)complete response.
Red = high risk, i.e. tumor unlikely to achieve a (near)complete response.

using the criteria detailed in the schematic drawings below:

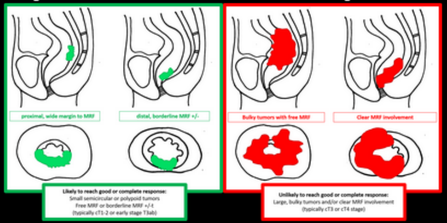

Click here for a larger representation of the image.

Please select the image that best reflects the current case:

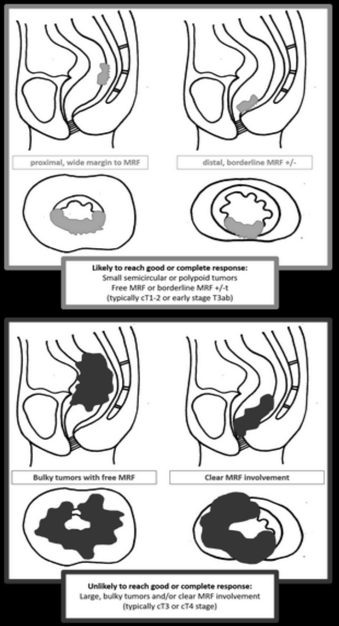

In this particular case, I found this scoring system...

- [ ] Easy to apply.
- [ ] Moderately easy/difficult to apply.
- [ ] Difficult to apply.

Panel D
